# Pathology and Therapeutics

**Published:** 1859-07

**Authors:** Emil Fischer


### PATHOLOGY AND THERAPEUTICS.

BY EMIL FISCHER, M.D.

### I.—*Anatomical and Physiological Researches on Dropsy consecutive to Typhoid Fever.* By DR. LEUDET.

The different kinds of dropsy following typhoid fever have been described by a great many pathologists; in France this subject has received less attention than in other countries. MM. Louis, Chomel, Forget, Guarin, and Piedagnel described only the œdema confined to the lower extremities. M. Martin is perhaps the only French author who has given an account of the anasarca and ascites following typhoid fever.

Partial œdema has been very well described by R. Virchow, (*Archiv für pathologische Anatomie*, tome ii. p. 401,) and by Magnus Huss, (*Statistique et traitement du typhus et de la fièvre typhoïde*, p. 205; Paris, 1855.)

Dr. Griesinger, of Tübingen, has given a very complete description of these dropsies (in *Virchow's Handbuch der Pathologie, etc.*, tome ii. p. 173, 1857.)

"There exists," says he, "a local dropsy consecutive to the coagulation of the blood in the veins, the cause and signification of which are evident. We meet, as a very rare complication of typhoid fever, in its second stage, an extensive dropsy of the cellular tissue and of the serous membranes. This dropsy is more common in certain epidemics; I have observed it, during an epidemic which befell a population suffering from dearth, in about one-fourth of the cases. The œdema supervened generally at the end of the third week, more rarely at the end of the second; it commenced sometimes in the face, at other times in the inferior extremities, spread rapidly over the whole body, and was often accompanied by more or less ascites. A small number of patients presented, in addition, a marked albuminuria. In most cases it was not very decided, or had already ceased. During this time the skin was warm and dry; a copious eruption of miliaria accompanied the beginning of the dropsical effusions, and persisted as long as they lasted; sometimes the pulse was at the same time slow and irregular. The internal organs showed no symptoms of disease. This dropsy lasted generally from six to ten days and disappeared mostly within a few days. It exercised no unfavorable influence upon the termination of the disease; in fact, only a small number of patients succumbed. The dropsy aggravated the prognosis only by the circumstance that it prolonged the period of convalescence."

M. Leudet had not seen a single case of this dropsy during nearly ten years of observation made in the hospitals of Paris; but having had charge, for about four years, of a division of the Hôtel-Dieu of Rouen, he had the opportunity of observing a certain number, and has reported seven cases of this kind in his memoir.

A careful comparison of his observations with those published by other authors enabled him to draw the following conclusions:—

The typhoid fever which is followed by dropsy is characterized in Rouen, as well as in Paris, by ulceration of the intestinal follicles. The serous infiltrations, whether confined to the lower extremities, or general, were of the nature principally described in Germany. They are rarely limited to the superior extremities or the face.

Sometimes the effusion takes place into the peritoneum; M. Leudet has observed no case of this kind; in one instance he met with a peritonitis.

The œdema is colorless and painless, and is, therefore, easily distinguished from erysipelas. It supervenes mostly without prodromic symptoms, and coincides often with some exacerbation of the fever, with sweats, an abundant eruption of sudamina, and an intense bronchitis. This latter coincidence has been particularly remarkable in Rouen, but it is not constant. In the cases observed by M. Leudet, as well as in the epidemic studied by M. Griesinger, the œdema showed itself, generally, in the second or third week of the disease, and disappeared in two or three weeks.

M. Leudet has been unable to discover albuminuria or lesions of the kidneys in any of his cases. These two morbid states have been observed, however, by

Rayer, Martin Solon, Finger, Frerich, Begbie, Christison, Johnson, Griesinger, Barthez and Rilliet.

The cause of this œdema, says M. Leudet, seems to be the constitutional weakness of the inhabitants of our city, and the very nature of the typhoid fever, which is more frequently accompanied by stomatitis, pleurisy, etc. than anywhere else. Besides, the fevers and inflammations have generally an adynamic character in Rouen. The anasarca is not a serious complication of the fever; it prolongs, however, the period of convalescence. The treatment consists of tonics exclusively. (*Archives Générales,* October, 1858, and *Gazette Hebdomadaire*, March 18, 1859.)

II.—*On the Existence of Albuminuria in Yellow Fever.* By Dr. Arnoux.

The attention of phathologists has been repeatedly directed to the existence of albuminuria in yellow fever. Dr. Arnoux, Physician-in-chief of the Naval Forces at Martinique, adds new and important testimony to what is known about this symptom. A report of his contains the following passage:—

" We will not conclude this general review of the diseases which have reigned during the last quarter of the year 1858, without referring to a case of sporadic yellow fever, the diagnosis of which was confirmed by the presence of albumen in the urine. As long as the disease followed its regular course, albumen was present in great abundance. At the decline of the second stage, it was perceptibly diminished, and disappeared completely as soon as convalescence was established. We have at the same time had an opportunity of making a comparative observation in the case of an artilleryman, who presented, at the moment of his admission to the hospital, decided symptoms of typhus icterodes, except that peculiar *facies,* which is a serious indication for the physician accustomed to observe these kind of affections. This experience has given still more weight to the pathognomic symptom of yellow fever pointed out by Dr. Ballot. The absence of albumen confirmed our first diagnosis, and sulphate of quinia, administered toward the end of the paroxysm, *promptly cured* a disease which had simulated a malady for which we unfortunately possess no remedy heroic enough." (*Gazette Hebdomadaire,* May 6, 1859.)

III.—*On the Use of Tannin in large doses in Albuminous Anasarca.* By Dr. Garnier.

Pure tannin, tannic or gallic acid, was at first prescribed in hemorrhages and diarrhœa, in pretty small doses, as it was considered a poison. Later, many practitioners recommended its use in nocturnal sweats, in diabetes, and in atonic diseases. Dr. Garnier, of Paris, was induced to try tannin in the albuminous anasarca consecutive to scarlatina, a disease which so obstinately resists ordinary measures. From the observations he has lately published in the *Archives Générales de Médecine,* it results that under the influence of large doses of this agent, the general infiltration of the cellular tissue, and the presence of albumen in the urine have simultaneously disappeared. Dr. Garnier gives the following conclusions at the end of his treatise:—

1. Tannin, in the dose of fifteen to thirty grains through the day, cures anasarca or œdema which has been passively developed, and coexist with albuminous urine. 2. Its curative action manifests itself by copious urine, which gradually assumes its physiological characters, by perspiration, loose stools, etc. 3. These symptoms appear on the second day of the administration of the tannin. 4. Given in solution of about three to eight grains, tannin does not derange the digestive organs. 5. The action of tannin seems to be primitively directed upon the fluids of the economy, the albuminous principles of which it coagulates and plastifies; its action upon the solids seems to be consecutive, tonic, and astringent.

The facts reported by Dr. Garnier are quite interesting; it is, however, important to remark, that tannin has been given only in cases of transitory albuminuria, following congestion of the kidneys, and that albuminous or granular nephritis is not at all in the question. This distinction should be made in order to appreciate therapeutic methods properly. (*Archives Générales*, January, 1859.)

### IV.—*On Urœmia.* By PROF. OPPOLZER.

The nature of uræmia is not yet agreed upon by pathologists. In reference to the changes of the blood in Bright's disease of the kidneys, we know only that the proportion of albumen and of the blood-corpuscles is diminished, while that of water and of urea is augmented. The accumulation of urea cannot, however, be received as the cause of the uræmic symptoms, as injections of urea in animals do not produce any perceptible disturbance, except an increased secretion of urine. Supported by experience, and many experiments on animals, in whom he injected carbonate of ammonia, ($NH_3$ $CO_2$,) Frerich advanced the view *that urœmia is owing to a decomposition of the urea accumulated in the blood, into* $NH_3$ $CO_2$.

Experience at the sick-bed teaches, indeed, that the opinion of Frerich, although having been frequently contested of late, is still to be sustained by a great number of cases.

Some object, that the $NH_3$ $CO_2$, in the alkaline matter vomited, is not secreted as such from the blood, but is formed only by the decomposition of the urea present in the contents of the stomach; but although this objection cannot be directly refuted, it is by no means applicable to the $NH_3$ $CO_2$ contained in the expired air. Prof. Oppolzer has repeatedly made the observation that in many cases of Bright's disease, where well-marked symptoms of uræmia made their appearance, so large an amount of $NH_3$ $CO_2$ was contained in the expired air, that in a solution of MgO, $PO_5$, through which the expired air was conducted, great quantities of triple phosphates were precipitated. In employing this method of investigation, it was, of course, necessary to ascertain first the absence of other sources of ammonia, such as inflammatory processes in the cavity of the mouth and throat, caries of the teeth, or a disease of the lungs in the suppurative state.

Moreover, Professor Oppolzer often observed in cases of Bright's disease, which had died of acute œdema of the lungs, and which were subjected to a post-mortem examination a short time after death, when putrefaction could not yet have commenced, that the serous fluid contained in the lungs smelled strongly of $NH_3$ $CO_2$. All these circumstances seem to prove that in a number of cases the urea accumulated in the blood is decomposed during lifetime into $NH_3$ $CO_2$, and that this decomposition occasions those symptoms which are believed to indicate uræmia. It is not known what causes this decomposition of urea, and whether a peculiar ferment is formed at the process; experience teaches so much, that with intercurrent, acute diseases, uræmic symptoms are apt to set in. Those opponents of the theory of Frerich, who admit the possibility of $NH_3$ $CO_2$ being developed in the blood, believe, nevertheless, that it must very quickly be eliminated again by the lungs, the skin, and alimentary canal, without giving rise to dangerous symptoms. They do not consider, however, that the material for $NH_3$ $CO_2$, the urea, is constantly formed out of the nitrogenous constituents of the blood. So much is certain, that when the vomiting of alkaline matter, and the secretion of $NH_3$ $CO_2$ by the lungs cease, the uræmic symptoms increase in a considerable degree.

In discussing Frerich's theory of uræmia, we have made the remark, that it can be accepted only for a certain class of cases; it is now the question, whether in those cases in which a decomposition into $NH_3$ $CO_2$ cannot be proved, clinical observation points out another change as the cause of uræmia? An accurate answer to this question is out of the range of present experience. We believe ourselves, nevertheless, justified in assuming, *that in a great number of cases the uræmic symptoms are connected with a serous infiltration of the brain and its membranes.* The author is supported in his opinion not only by very numerous autopsies, but also by the phenomenon, very often observed during lifetime, that in individuals suffering of greatly developed dropsy, the latter rapidly decreases without there being a correspondent increase in the secretion of urine; at the same time the jugular veins become turgid, and decided symptoms of uræmia are developed; the post-mortem examination reveals a serous infiltration of the brain.

Uræmic symptoms are pretty frequently observed in acute Bright's disease, where the greatest part of the parenchyma of the kidney, infiltrated with exudation and gelatine cylinders, allows the excretion of but a small quantity of urine; most generally, however, they occur in the chronic form of the disease, where the destruction of the parenchyma of the kidneys is so far advanced that the function of the organ is completely suspended. The symptoms ascribed to uræmia are very various; we not only include among them the cerebral symptoms, characterized by stupor, sopor, coma, convulsions, in short, eclampsia, but also the nervous symptoms already mentioned, and all the different processes of exudation occurring during Bright's disease.

The uræmia of acute Bright's disease is in many respects different from that accompanying the chronic form of the disease; it usually makes its appearance suddenly, with an attack of eclampsia, and is but rarely preceded by headache or stupor. The eclamptic attacks succeed each other quickly; in most cases consciousness is restored during the interval; the intervals between the later attacks become gradually shorter, until death occurs during an eclamptic fit.

In another series of cases the author observed, after one or several eclamptic attacks, a sudden increase of the secretion of urine which, at the beginning, contained still a large quantity of albumen; but in a short time the urine became clear, the exudation cylinders disappeared entirely, and albumen was present only in traces; at the same time consciousness returned, and convalescence progressed so rapidly that within a few days the patients could be considered cured.

Professor Oppolzer is of the opinion that this sudden change of the disease is owing to an altered state of innervation of the capillary vessels of the kidneys, which is probably produced by the eclamptic fit, for it can hardly be supposed that the process of exudation in the kidneys could suffer so rapid a change that the exuded matter would be suddenly resorbed or eliminated. This view of the influence of the nervous system upon the urinary secretion appears, independently of the process of exudation, a great deal more plausible, if we observe the great influence of the nervous system on the urinary secretion in general.

Professor Oppolzer has seen several cases of persistent alcalescence of the urine, where the latter became at once clear, and of acid reaction, after a catheter had been introduced into the bladder.

The cerebral symptoms of uræmia in chronic Bright's disease rarely make their appearance suddenly; they commence often with vomiting of alkaline matter, headache, and stupor, upon which delirium, convulsions, and death may follow; or the stupor turns into sopor and coma, and death occurs without a previous eclamptic attack.—(*Wiener Spitalszeitung*, 1859, 3, and *Medizinische Central Zeitung*, March 23d, 1859.)

### V.—*A Case of Splenic Leucocythœmia.* By PROF. OPPOLZER.

H. E., forty-five years of age, mother of five children, had, without any known cause, a chill in the month of August, 1857. The attack recurred the next day, then at the end of fifteen days; from that time the attack was repeated daily for eight days. The patient perceived, at the same time, that she had a tumor in her left hypochondrium as large as a fist.

In January, 1858, this tumor had acquired the size of a child's head, and had become painful. The patient felt well for six weeks, in February and in March, then the attacks of fever occurred frequently, and the spleen became the seat of a more intense pain. Sulphate of quinia was administered; the paroxysm returned then several times a day, without regularity; the patient had also an obstinate diarrhœa. On the first of April she entered the clinic of Professor Oppolzer.

The lymphatic glands of the neck and of the groin were somewhat swelled, the liver was very voluminous. The spleen extended from the seventh rib to the crest of the ilium and to about one inch above the symphysis of the pubis; above the umbilicus it reached the median line, while below this point it stretched beyond it; posteriorly it occupied the lumbar region, which felt very hard on palpation. The spleen had a smooth surface, and its blunt border presented several indentations toward the inferior extremity of the tumor; it was the seat of continual pain, which became more intense during the cold stage of the febrile paroxysm. Digestion was good during the apyretic state; the urine was slightly albuminous; the blood presented a buffy coat of remarkable whiteness, and below it white granules of the size of a millet-seed formed exclusively by white corpuscles. The clot was voluminous and red.

The febrile paroxysm and the pain in the spleen persisted in spite of the administration of forty-two grains of quinia within four days, and of several applications of leeches to the splenic region; the blood became gradually richer in white corpuscles. Fowler's solution produced no improvement.

On the nineteenth day after the admission of the patient, the spleen was found still augmented in size. Large doses of sulphate of quinia afforded a slight amelioration; salicin, administered in a dose of two scruples, produced no effect. Afterwards the patient was attacked with diarrhœa which yielded to the administration of extract of colombo, and of tannin. Ultimately, a thrombosis of the saphenous and crural veins was noticed. The patient died on the fourteenth of July, after a short agony.

M. Folwarczny made an analysis of the blood after Scherer's method. He found formic acid in all his specimens, lactic acid in the blood of the capillaries, of the median and supra-hepatic veins, and of the aorta; tyrosin and leucin in the blood of the aorta and of the capillaries; traces of uric acid in that of the jugular vein. The relative proportions of water and of solid constituents, of organic and inorganic matters, did not differ materially from those found in the normal state.

The blood contained, besides a great quantity of white corpuscles, free nuclei, quite analogous to those which are found in swelled lymphatic glands.—(*Wiener Allgemeine Medizinische Zeitung*, 1858, 29 and 32, and *Prager Vierteljahrschrift*, 1859, I.)

VI.—*On Nervous Vertigo*, (Prize Essay.) By DR. MAX SIMON, of Aumale.

The following is a short abstract of the treatise of Dr. Max Simon "on nervous vertigo," which obtained the prize from the Académie de Médecine of Paris.

After a long introduction, in which the author dwells, among other things, upon the fact that vertigo may sometimes be a mere neurosis, independent of material alterations of the brain or of other organs, the author proceeds to the classification of the varieties of the disease. He distinguishes (1) the *idiopathic* vertigo, that is, a mere neurosis, where neither in the nervous system nor anywhere else a statical or dynamical disturbance exists, upon which the vertigo may depend; and (2) the *sympathetic* vertigo which is owing to a functional disorder of some other organ, except the brain. The author agrees with P. Frank in defining vertigo as a sudden and distressing sham-turning which seems to seize one's own person and the objects surrounding it, no matter whether they are in rest or in motion, whereby the body staggers and threatens to fall.

(1.) The *idiopathic* vertigo occurs, like all neuroses, periodically, with complete intermissions between the attacks, during which not the slightest inclination to an attack is manifested. It is of different intensity, and occurs in various forms. It may seize the patient while standing, lying down, or while raising himself up from a stooping position. In the higher degrees of the affection, where either the cause acts more intensely or the nervous system is more impressible, the symptoms of anxiety are added, the sight becomes dim or is lost entirely, hemiopia or diplopia supervene, and the patient falls, but does not lose consciousness, or if he happens to lose it, the cause of it is anxiety rather than the functional disturbance of the brain with which the vertigo is connected. The same is true in regard to the confusion of thought which is sometimes observed, particularly in hypochondriac patients, and after strong emotions. Also in sea-sickness, disturbance of the senses is frequently observed. With such intensity of the affection the pulse too is accelerated without a corresponding increase of temperature; not uncommonly nausea and vomiting supervene, the latter, however, being more frequent in dyspeptic vertigo.

(2.) The *sympathetic vertigo of dyspepsia* does not differ from the other in regard to its symptoms; it is only considered separately on account of the frequency with which it accompanies those undefined disorders of digestion which are called dyspeptic. Its disappearance on the cure of dyspepsia proves its dependence on the same. Whether it is caused by too great an amount of acid, as Dr. Sandras believes, the author does not attempt to decide; it is at any rate easily cured by alkaline remedies.

(3.) *The vertigo in consequence of hypochondriasis, excess "in venery," and spermatorrhœa.* In hypochondriac patients vertigo originates partly from the disorders of the alimentary canal, so common in hypochondriasis, and partly depends upon the whole condition of the nervous system. The same is true in regard to many cases of sexual excess which may at first influence the stomach, and through the latter the brain. But at a later period it may also be produced by anæmia. In the earlier stages of the affection anæmia cannot, of course, be regarded as an explanation. In this variety of the disease we may consider the nervous excitement attending each coitus likewise a cause, which may operate just as much here as in abstinence.

(4.) *The vertigo of convalescence* depends only partly upon anæmia, partly upon the dynamic change of the nervous system caused by the altered crasis of the blood. That this is the case is evinced by the fact that vertigo does not occur equally after all diseases producing anæmia, but particularly after malignant fevers, such as typhus, being associated in this case often with mental disturbances.

(5.) *The vertigo at sea, sea-sickness;* the manner in which sea-sickness makes its appearance, as well as the influence which mental excitement sometimes has upon the disappearance of the disease, seem to indicate that it belongs to the varieties of nervous vertigo, and that it differs from the rest only by its violence.

The course of vertigo is various; the attacks are either short and often repeated, or they last longer and keep longer intervals; the disposition to the affection is either transient or lasts for life. In general, the vertigo disappears as soon as the cause of it is removed, or a short time after.

The prognosis is always favorable; those who entertain the opposite opinion, confound the vertigo with affection of the brain which may simultaneously exist.

In regard to differential diagnosis, it is necessary to distinguish the nervous vertigo from that resulting from anæmia, plethora, and organic diseases of the brain. Without regard to the well-known symptoms of anæmia, the anæmic vertigo differs from the simply nervous one by the frequent occurrence of syncope and tinnitus aurium as premonitory symptoms. The plethoric vertigo is characterized by the general symptoms of plethora; it is in this variety particularly that headache and a feeling of fullness in the brain are experienced, together with the various disturbances of the senses. The vertigo in diseases of the brain is distinguished by symptoms arising from the nervous system, especially, however, by the persistent headache. At the commencement, it is often difficult to recognize its nature, particularly if the stomach is sympathetically affected.

To these varieties belongs the syphilitic vertigo, which probably owes its origin, in most cases, to exostoses. Also the vertigo observed in cases of lead-poisoning finds its place here; it is easily discerned. With epilepsy nervous vertigo cannot easily be confounded.

In considering the etiology (1) of idiopathic vertigo, the author enumerates all the different exciting causes without saying a word about the internal causes common to the disease in general. They are quick motion of objects, or of one's own person, a glance into an interminable space, no matter whether the patient is upon the level ground or upon a high elevation, hunger, coffee, tea, beer, particularly if containing much hops, tobacco, hemlock, and vapor of burning coal. Hereditary disposition, mental excitement, sex, and age, are thoroughly considered. (2) About the causes of the sympathetic vertigo the author gives also but deficient information. In reference to sea-sickness he mentions that it is caused by the movements of the ship, by the glance into an interminable space, by anxiety, and by imitativeness. Children are, therefore, the less liable to sea-sickness the younger they are, because the mentioned causes do not act in their case, except the motion of the ship, which, however, is hardly to be taken into consideration in the case of very small children, as it operates but indirectly.

In regard to the treatment, the physiological vertigo, as it could be called, does not demand any medical interference; it yields to time and habit. The idiopathic vertigo requires regulation of the diet; the patient should not fast too long; his bowels should be kept regular; aromatics should be tried; also mental influences are of value: it is, however, difficult to say of what kind they ought to be. During the attack itself bodily and mental rest is to be enjoined; in case of fainting, we may have recourse to stimulants. The dyspeptic vertigo demands alkalies to correct the acidity of the stomach commonly present; they are, however, also otherwise useful. Other disorders of the stomach are to be met with the means appropriate to them.

The hypochondriac vertigo is to be treated like the idiopathic variety; at the same time the hypochondriac temper should be regarded. The same rules hold good for the vertigo from sexual excess; in this variety lupulina may be used with great advantage, as it allays the excitement which is the cause of the affection.

The vertigo, after serious febrile disease, rarely necessitates any treatment. Duchenne and Wedl have obtained favorable results from the use of cinnabar. In regard to vertigo after intermittent fever, it remains doubtful whether it is a symptom of miasmatic poisoning, or whether it is produced by the use of quinia.

and down upon deck, to eat moderately, not to look, if possible, upon the objects in motion, and to smell and to take stimulants. As soon as vomiting commences, the horizontal position should be kept. The arthritic vertigo, finally, demands, if it really exists, the remedies usually employed in arthritis.—(*Mém. de l'Acad. de Méd.*, XXI., 1858, and *Schmidt's Jahrbücher*, 1859, I.)

VII.—*Contributions to the History of Nervous Diseases of Syphilitic Origin.* By DR. GJÖR.

The description of these diseases is based upon the accurate observation of thirty cases. Fourteen of the patients were less than thirty-five years of age, eleven were from thirty-five to forty years, and only one individual was more than forty-five years old.

In the great majority of the cases, the invasion of the disease was preceded by distinct prodromic symptoms; they consisted of pain in the lumbar region or in the extremities, or of an obstinate headache, with nocturnal exacerbations.

Most frequently the characteristic signs were of a paralytic nature; the symptoms which accompanied the commencement of the paralysis were in general not very serious, and rather fugacious; fifteen times attacks in the form of apoplexy occurred, but were of but little intensity; five times only these attacks gave rise to a complete loss of consciousness, and consecutively to weight in the head; twice the loss of consciousness was associated with convulsions; in two cases the apoplectiform attack occurred twice. Of these fifteen cases, there were only four in which the paralysis supervened suddenly, and without the health having been seriously impaired; in the eleven other cases it developed itself imperceptibly.

In half of the cases hemiplegia was noticed; eight times, paraplegia; twice, facial hemiplegia; in three cases, paralysis confined to one extremity; in two, a general weakness of the movements of the four extremities; in nine, anæsthesia; and in two cases, hyperæsthesia. The paralysis of the extremities was accompanied, in several cases, by paralysis of the sphincters, and in four cases by amblyopia, with dilatation of the pupil.

The interval which separated the first symptoms of constitutional syphilis from the paralytic attacks, was scarcely obvious in two patients; several months to a year, in eleven; one to five years, in eight patients. In the rest of the cases the appearance of paralytic symptoms was retarded still longer.

It seemed that the patients who had suffered from several relapses of the symptoms of constitutional syphilis were not any more exposed to the nervous diseases than those in whom these symptoms had been observed only once; the number of cases of the first category is, in fact, only ten in the statistics of Dr. Gjör; he observes, however, that the number of his cases is not sufficient to deduce from them unobjectionable conclusions.

The treatment of the nervous diseases of syphilitic origin has not given, up to the present time, very satisfactory results. Of the thirty patients of Dr. Gjör, only five were cured; in twelve a more or less decided amelioration was obtained; in six cases, no change took place, and seven times the disease had a fatal termination.

Dr. Gjör employed particularly the iodide of potassium, and often combined with it strychnia, or the preparations of arnica; this remedy has afforded him the most advantageous and prompt results. Mercury was employed in five cases, and was not successful in a single one. Dr. Gjör tried syphilization several times; in one case only it produced a rapid cure, in the six other cases it was not followed by any improvement: in all syphilized patients the state of the general health was, however, much improved.

The three autopsies reported by Dr. Gjör prove, at least, that nervous diseases of syphilitic origin are not always owing, as was formerly supposed, to exostoses situated in the cavity of the cranium or of the spine; in one case, Dr. Gjör found softening of the brain; in the two others no lesion of the nervous centres could be detected.—(*Schmidt's Jahrbücher*, 1859, CI., p. 929; from the *Norck Magazin*, XI., p. 794.)

VIII.—*On the Treatment of Chronic Organic Diseases of the Heart.* By PROFESSOR LEBERT.

The treatment of chronic organic diseases of the heart offers one of the most difficult tasks to the practicing physician; it is nevertheless certain that a proper and thorough treatment of these diseases may do a great deal toward alleviating the condition of the patients, and toward prolonging their lives. Prof. Lebert advises especially great care with blood-letting, purgatives, and all debilitating measures in the treatment of organic diseases of the heart. In valvular disease he employs venesection but very rarely. If in the course of the disease an acute inflammation, in the form of pericarditis or endocarditis, occurs, it is often useful to abstract blood to the amount of six ounces by means of cups or leeches; if, after this, an energetic treatment is still necessary—much advantage will be obtained from the application of a large blister, and the endermatic use of half a grain of morphia daily. The author's observations on the use of digitalis we may omit as generally known. Most allied to digitalis, in regard to its therapeutic effect, is aconite. Although it acts less heroically and more slowly than digitalis, and does not decidedly diminish the frequency of the pulse, aconite is nevertheless a remedy which is capable of lessening considerably the dyspnœa, palpitation, and the various subjective symptoms of the patient, even the tumultuous excitement of the heart. A very important point to be considered in the treatment of advanced diseases of the heart, is the general cachexia and debility of the patient, gradually developed. Lebert has examined for several years the muscular structure of diseased hearts, in order to see how far the gradual decrease of the functional capacity of the same depends upon changes in the muscular fibre itself. From these investigations it results that very frequently a small degree of fatty degeneration of the primitive cylinders of the muscle of the heart exists, even in cases where the color and consistency of it does not indicate the fact. The gradual increase of general debility and of the local weakness of the heart, the progress of anæmia and hydræmia, lead to the question, what effect tonics, especially iron, would have in organic disease of the heart.

The better the patients are nourished (with avoidance of strongly stimulating food) the longer they resist the evil influence of the disease; the patient should, Against sea-sickness no reliable prophylactic is known. It is well to walk up therefore, not be restricted to a vegetable diet, but a moderate amount of animal food should be allowed with it. Of beverages, tea, coffee, alcoholic liquors, and wine in large quantities ought to be avoided, whereas infusion of cocoa, or decoction of roasted acorns, are very appropriate, particularly for breakfast. Light beer, or small quantities of old wine mixed with water, may be allowed at the table. Besides an analystic diet, the use of iron is indicated, particularly in the later anæmic and cachectic period of the disease. Lebert recommends especially iron reduced by hydrogen, (gr. ij at each meal,) or twenty to twenty-five drops of the tinctura ferri pomati, if necessary, in connection with equal parts of tincture of aconite. The tartrate of iron and potassa (three to five grains three times daily) is a very useful preparation, and as the effect of iron is perceptible only then, when its use is persisted in for a long time, it is necessary, occasionally, to make a change in the preparation employed. The author has not derived much advantage from the application of blisters, setons, and moxas.—(*Wien. Medizin. Wochenschrift*, 1858, No. 51, and *Mediz. Neuigk.*, April 2, 1859.)

IX.—*Observations on the Diagnosis of Diseases of the Pancreas.* By PROF. OPPOLZER, of Viènna.

The most important diseases of the pancreas, evinced by anatomical changes, are scirrhus, atrophy, hypertrophy, acute and chronic inflammation. *Scirrhus of the pancreas* is much rarer than *scirrhus around the pancreas* (scirrhus circa pancreatem;) its diagnosis is therefore not easily established, and this difficulty is increased by the circumstance that the evacuation of fæces containing fat is not peculiar to diseases of the pancreas, as was formerly believed, but may also occur in affections of the mucous membrane of the duodenum, and that the digestion of amylaceous matter may be hindered without disease of the pancreas being the cause of it.

*Atrophy of the pancreas* has recently attracted much attention by its occurrence in diabetic patients, and by Bouchardat ascribing to these two diseases the relation of cause and effect. It is proved that atrophy of the pancreas may be also produced by impediments in the secretory duct, or at its mouth, such as obstruction of the duct of Wirsung by foreign bodies, further by biliary calculi, cancer, and the cicatrix of a perforating ulcer near the diverticulum Vateri. It is, however, impossible to diagnosticate atrophy of the pancreas from the symptoms which accompany the disease, such as pain in the region of the pancreas, fatty stools, and emaciation.

*Enlargement of the pancreas* may take place in consequence of the deposit of fatty matter, whereby the acini become compressed, and the whole organ is converted into a mass of fat. In this affection also no characteristic symptoms are known, not considering that it is quite impossible to diagnosticate a lipoma within the peritoneum.

The occurrence of *acute inflammation* of the pancreas has been wholly denied. Oppolzer himself, however, saw the organ in one case swelled, reddened, and with exudation between the acini, as in parotitis. The patient suffered from oppressive pain in the pit of the stomach, and tried to obtain relief from it by taking brandy and pepper. This was followed by vomiting and increase of the pain. The vomited matter consisted of mucus and bile. The patient had a violent fever, very frequent pulse, and cold extremities, his face was shrunken, and he suffered from obstinate constipation. There was no blood vomited; and the case was diagnosticated as one of perforating ulcer of the posterior wall of the stomach. On the third day after being admitted into the hospital, the patient died, and the post-mortem examination proved the stomach to be healthy; around the pancreas, however, and between the layers of the mesentery, a large effusion of blood was found; the pancreas itself was enlarged to double its size, of a dark-red color, and contained an exudation colored with blood between the acini.

*Chronic inflammation* of the pancreas was formerly diagnosticated very frequently; but it was usually confounded with perforating ulcers of the stomach. Chronic inflammation of the pancréas may, however, coexist with gastric ulcer, or with ulcer of the duodenum, and is then a secondary affection, the ulcer of the stomach or of the duodenum having formed adhesions with the pancreas. Prof. Oppolzer has observed a case of this kind; the patient, a girl, had suffered from a perforating ulcer of the duodenum, and on post-mortem examination adhesions between the ulcer and the pancreas were found.

For some time it was considered dangerous to suppress salivation produced by a mercurial treatment, as it was thought that such a measure would give rise to an undue secretion of the pancreatic fluid and to diarrhœa. Nothing, however, is known concerning the sympathy between the salivary glands of the mouth and the pancreas, as to whether diseases of the pancreas usually cause diarrhœa.

In regard, finally, to determining the position of the pancreas by percussion, it would not be impossible to feel it in the abdominal cavity, if the abdominal parietes were relaxed, and under normal conditions; but for estimating the size of the pancreas, and for drawing conclusions in regard to its diseases, there is exceedingly little to be gained by percussion.—(*Allg. Wiener Medizinische Zeitung*, and *Medizinische Neuigkeiten*, April, 1859.)

X.—*Memoir on the Oxalate of Lime in the Sediments of the Urine, and on the Gravel and Calculi of Oxalate of Lime.* By M. Gallois. (Read before the *Académie des Sciences* of Paris, April 4th, 1859.)

The following propositions contain the substance of M. Gallois's memoir:—

1. Oxalate of lime is a substance which may be found transiently in the urine of the healthy individual, at all ages and at all periods of life.

2. It makes its appearance in more or less considerable proportions, especially under the influence of certain aliments, and probably of certain medicines.

3. Oxalate of lime is frequently met with in the urine of the sick, but its excretion does not constitute a disease by itself. Oxaluria is thus not a morbid entity, but only a symptom common to very different affections. Nevertheless, it is proper to state that oxaluria is observed oftener in spermatorrhœa, and in certain diseases of the nervous system, especially in dyspepsia.

4. There is a body which very frequently accompanies oxalate of lime in the urinary sediments, as well in gravel as in calculi; this body is crystallized uric acid.

5. The very common coexistence, in the urine and urinary concretions, of uric acid and oxalate of lime, seems to me to explain the formation of the oxalate of lime within the organism.

6. The relation which pathologists have endeavored to establish between oxaluria and diabetes, is not admissible.

7. Oxalic acid (and consequently oxalate of lime) seems to be derived from uric acid, and ought to be considered as an advanced degree of oxidation of the latter body, or of the elements which serve to constitute it; thus whenever there is uric acid in the economy, or the elements which are necessary to form it, oxalic acid can be produced under the influence of a more advanced oxidation which takes place in the blood.

8. Oxaluria demands, ordinarily, no other treatment than that of the physiological or morbid condition with which it is associated. A great variety of remedies have been advised in the treatment of oxaluria:—First, abstinence from aliments and medicines containing oxalic acid; second, the use of small doses of nitromuriatic acid in a bitter and tonic infusion, of nitrate of silver (in gravel,) of colchicum (in certain cases,) or of phosphate of lime, etc.

9. For my own part, I have found that the alkaline mineral waters constitute the most efficacious means to prevent the excretion of the oxalate of lime, particularly if it is accompanied by a deposit of uric acid, a condition which seems to me the most frequent of all.—(*Archives Générales*, May, 1859.)

XI.—*Asbestus as an Irritant Application to the Skin.* By DR. V. KLETZINSKY.

The study of a chemically indifferent but powerful mechanical irritant to the skin would certainly be of great service in regard to the treatment of cutaneous diseases and ulcers. An irritant of this description, which has been either unknown or unnoticed until the present, is asbestus, reduced to a very fine powder. In order to prepare it, common asbestus (as found in the market) is boiled with an excess of dilute hydrochloric acid, collected afterwards upon a percolating cloth of dense texture, and washed thoroughly with water; the pap of asbestus is put, still moist, into a mortar of serpentine-stone or porcelain, mixed well with water, and washed. The deposit of the slime of asbestus is then dried and kept for use. The chemical composition of this silicate of magnesia excludes the possibility of chemical action being concerned in the therapeutic effect produced by it; and with no other substance of equal therapeutic applicability is this so much the case. Dr. V. Kletzinsky made the accidental discovery that this chemically inert substance produced mechanically a very powerful effect, which he explains by the circumstance that the very fine and short fibres of the asbestus act in the manner of the hairs of caterpillars and of the pubescent glands of certain plants, by hooking themselves into the pores of the skin, or into the raw surface, thereby producing a permanent and purely mechanical irritation. The author thinks it probable that the application of asbestus to ulcerous and diseased parts of the skin would produce in some cases a very favorable result, which, with the use of other remedies, would have been ascribed to chemical action. The experiment of applying asbestus in the proposed manner would be at any rate serviceable, in so far as it would enable us the better to distinguish whether the curative effect of certain remedies is owing to mechanical or chemical agency.—(*Zeitschrift für Praktische Heilkunde;* Wien, 1859, 6.)

### XII.—*Easy and Certain Cure of Facial Neuralgia.* By DR. BURDACH, of Luckau.

Dr. Burdach recommends corrosive sublimate as a specific, never-failing remedy, in cases of facial neuralgia. He has used it for more than thirty years, and always obtained a prompt and permanent cure, no matter how severe a form the disease had assumed. The formula he employs is the same which he recommended in *Hufeland's Journal* for 1826 and 1830, in the treatment of rheumatic gout; it is the following:—

℞.—Liquor. Hydrarg. Bichlorid. corrosiv. (Pharmac. Boruss.) ℥jss;
Vini Semin. Colchici, ℥ss.—M.
S. Thirty to sixty drops every two hours.

Cases requiring the latter dose were extremely rare. (The Liq. Hydrarg. Bichlorid. corros. of the Prussian Pharmacopœia contains corrosive sublimate and hydrochlorate of ammonia, one grain of each to the ounce of water.) Each dose of the medicine should be followed by a draught of the decoction of the Species ad Decoctum lignorum; (the species ad decoct. lignor. consists of Guaiacum-wood, two parts; Lappa, and Saponaria, one part of each; Liquorice-root and Sassafras, half a part of each. One ounce of this mixture is used to a pint of water.) There is about one-thirtieth to one-fifteenth of a grain of sublimate given in each dose, a quantity which is generally well borne by the patients. In order to assist the cure, Dr. Burdach sometimes ordered the local application of veratria ointment, but in the generality of cases it could be dispensed with, as the sublimate acted promptly enough without it. In very sensitive patients, acetic acid, chloroform, or tincture of opium, might be added to the given formula; such an addition, however, is not to be recommended.

To obtain the prompt action of the remedy it is absolutely necessary to give it in fluid form, and at the intervals prescribed above, for in the form of pills it seems to exercise but little control over the disease.—(*Medizinische Central Zeitung*, December, 1858.)

### XIII.—*Gunpowder, a Remedy for Toothache and for Scabies.* By M. LAFFONT.

All remedies are good, if they cure. M. Laffont recommends his patients to put a teaspoonful of powder into a piece of fine linen, and to apply this little bag to the painful tooth. He has employed this remedy in twenty cases. All the patients told him that it provoked, but only for a few instants, a slight pungent heat in the mouth, but that the toothache completely disappeared.

M. Laffont learned from a traveler, that gunpowder was much used in the colonies against scabies. Three parts of powder, incorporated with ten parts of molasses, constitute a mixture, which is rubbed well on the whole body; the next day a bath is taken in water impregnated with soap, and the cure is finished. M. Laffont has used this friction in three cases of scabies, and has cured them radically.—(*Journal de Médecine de Bordeaux*, October, 1858.)

XIV.—*On the Therapeutic Action of Solanin, and of Dulcamara.* By PROF. CAYLUS, of Leipsic.

In order to examine into the facts already known in reference to the physiological and therapeutic action of dulcamara, and of its active principle, solanin, Prof. Caylus has undertaken a series of experiments which have led to the following conclusions:—

Solanin and dulcamara belong to the class of acrid narcotics, as far as they exert a paralyzing influence upon the medulla oblongata, and an excitant action upon the nerves; they cause death by producing paralysis of the respiratory muscles, their action being analogous to that of conicin, and of nicotin. They distinguish themselves, however, essentially from these substances, by not augmenting the sensibility of the cutaneous nerves, and by not exercising an instantaneous action upon the stomach and alimentary canal.

They differ from atropin, daturin, and hyosciamin, by the absence of delirium and stupor, of dilatation of the pupils, and of paralysis of the sphincters; from atropin in particular, by not producing pneumonia.

These substances possess therapeutic virtues in the spasms, and states of irritation of the respiratory organs; simple spasmodic cough, hooping-cough, and spasmodic asthma.

Their therapeutic action in certain diseases depending upon a dyscrasia of the blood, such as gout, rheumatism, constitutional syphilis, and perhaps also in certain chronic diseases of the skin (acne, eczema, ecthyma, impetigo,) may be owing to an increase of the excretion, by the veins, of certain constituent parts of the blood, and not to an excitation of cutaneous activity.

Contrary to the opinion generally entertained, solanin and dulcamara can be given without danger in inflammatory states of the stomach and alimentary canal, as they exert no action on these organs.

Inflammation of the respiratory passages does not offer a contra-indication to the use of solanin and dulcamara in the diseases of these organs; but if there is inflammation of the kidneys the contra-indication exists.

The medium dose for an adult is about one-sixth to three-quarters of a grain of acetate of solanin, a substance which Prof. Caylus prefers to the pure alkaloid on account of its greater solubility. The most convenient form in which to administer it is that of pills, the solution of the salts of solanin having a very disagreeable taste.

The extract obtained with alcohol, and afterwards washed with water to remove the alcohol, is preferable to the aqueous extract generally employed; it contains fewer mucilaginous substances and indifferent extractive matters than the latter, is more concentrated, and its dose can be more accurately determined.—(*Reil and Hoppe's Journal für Pharmako-dynamik*, and *Archives Générales*, March, 1859.)

XV.—*Employment of Iodoform as an Anæsthetic Agent.* By M. E. FRANCHNO.

The local anæsthetic action of iodoform has been repeatedly pointed out of late; some treatises published on this subject (*Bulletin de Thérapeutique*, January, 1857, and *Union Médicale*, October, 1857,) have induced M. Franchno to investigate whether the same agent could not be employed by inhalation. It is known that iodoform is not, like chloroform, a liquid which evaporates at a low temperature; it fuses only at 115°, and is sublimed at a little higher temperature; and although it disengages vapors at the common temperature of the air, as its saffron-like odor proves, it was not expected that inhalation under these circumstances would produce any sensible effects. The results announced by M. Franchno are thus of a nature to surprise us; but as it would be too great a skepticism to put them in question we will recapitulate them in a few words.

The apparatus employed by the author consists of a hollow ball containing a sponge sprinkled with iodoform, and provided with two openings; to one of them a muff is adapted, to be applied to the mouth; to the other a tube of the diameter of the trachea, destined to give passage to the air. Two grammes of iodoform suffice, generally, in animals, to produce anæsthesia in one and a half to two minutes.

The phenomena which reveal the action of the iodoform can be divided into two periods, analogous to those which have been established for most anæsthetic agents: in the first, agitation, contraction of muscles, acceleration of respiration and of circulation take place; in the second, calm, relaxation of the muscles, respiration somewhat slower, never stertorous; no disturbance of the circulation, complete anæsthesia which lasts four to five minutes after the cessation of the inhalation, then prompt restoration to the normal state.

M. Franchno has administered these inhalations only to animals. In regard to the question whether analogous results would be obtained in man we remain in doubt, as the three experiments reported by the author have been made on very small animals—a hen, a pigeon, and a rabbit; we know in addition to this, that rabbits are excessively sensitive to the action of anæsthetic agents.—(*Gazzetta Medica Italiana Stati Sardi*, 1858, 28, and *Gazette Hebdomad.*, April 8, 1859.)

XVI.—*On the Use of Alkalies as a Means of Obtaining the Extractive Principles of Vegetables.* By M. DANNECY, of Bordeaux.

M. Dannecy found that in the treatment of intermittent fever in which sulphate of quinia proved to be inactive, a number of empiric preparations were successfully employed, in which quinia was invariably combined with carbonate of potassa. This clinical result induced M. Dannecy to investigate in what the action of the alkaline carbonate consisted, and he came thus to the conclusion, that the alkalies (potassa or soda) were the most powerful adjuvants in obtaining the extractive principles of vegetables. He proposes, therefore, to add a small quantity of these substances to the water, in making pharmaceutical preparations, in order to increase the activity of the latter.

Cinchona bark treated in this manner furnishes extracts which have but little taste, and M. Dannecy believes that this circumstance would render them preferable to the ordinary extracts, particularly in the treatment of diseases of children.

For those vegetables which contain astringent principles among their elements, the addition of an alkali presents another advantage of great importance; it prevents, during the evaporation of the liquids, the formation of the body called *apotheme*, which is believed by pharmacologists to be the result of the oxidation of the extractive principle.

The preparation of the extract of rhatany, which offers this phenomenon in so high a degree, is protected from it completely by the addition of a small proportion of alkali to the water which serves to prepare it; and the evaporation in the open air does not furnish the smallest quantity of this insoluble principle which, in the preparation of the extract by the ordinary method, diminishes so sensibly the proportion and the quality of the extract soluble in the cold.

According to some experiments made on nux vomica and cinchona, M. Dannecy is inclined to believe that the process of extraction by the alkalies is destined to become a prompt and economical means to obtain not only strychnia and quinia, but also other immediate principles which have not yet been isolated. —(*Bull. de Thérap.*, and *Journal de Pharmacie et de Chimie*, March, 1859.)

### XVII.—*Antidote to Phosphorus*. By MM. ANTONELLI ET BORSARELLI.

Poisoning by phosphorus has become frequent, since the lucifer matches which contain this dangerous substance have been introduced into general use. Numerous experiments have proved to MM. Antonelli and Borsarelli:—

1. That in poisoning by phosphorus or by substances containing this metalloid body, it is above all necessary to avoid the use of fatty matters; as, far from opposing the action of phosphorus upon the organs, they only augment its energy and facilitate its diffusion in the economy.

2. That calcined magnesia, suspended in boiling water and administered in great quantity, is the best antidote, and at the same time the most convenient purgative to facilitate the elimination of the toxic agent.

3. That in cases of poisoning by phosphorus, in which dysuria is present, the use of acetate of potassa is of great service.

4. That the mucilaginous drinks administered to the patient should be prepared with boiling water, in order to have them contain the least possible quantity of air.—(*Union Médicale*, 1859, No. 10.)

